# The Therapeutic Role of PNU-74654 in Hepatocellular Carcinoma May Involve Suppression of NF-κB Signaling

**DOI:** 10.3390/medicina58060798

**Published:** 2022-06-14

**Authors:** Min-You Wu, Chi-Chih Wang, Ya-Chuan Chang, Chia-Ying Yu, Wen-Wei Sung, Chih-Jung Chen, Ming-Chang Tsai

**Affiliations:** 1 School of Medicine, Chung Shan Medical University, Taichung 402, Taiwan; wuburton123@gmail.com (M.-Y.W.); bananaudwang@gmail.com (C.-C.W.); raptor7037@gmail.com (Y.-C.C.); cyyu2015@gmail.com (C.-Y.Y.); flutewayne@gmail.com (W.-W.S.); 2 Division of Gastroenterology and Hepatology, Department of Internal Medicine, Chung Shan Medical University Hospital, Taichung 402, Taiwan; 3 Institute of Medicine, Chung Shan Medical University, Taichung 402, Taiwan; 4 Department of Urology, Chung Shan Medical University Hospital, Taichung 402, Taiwan; 5 Department of Pathology and Laboratory Medicine, Taichung Veterans General Hospital, Taichung 407, Taiwan

**Keywords:** PNU-74654, NF-κB, cyclin A, apoptosis, hepatocellular carcinoma

## Abstract

*Background and Objectives*: PNU-74654, a Wnt/β-catenin inhibitor, has reported antitumor activities; however, the therapeutic potential of PNU-74654 in hepatocellular carcinoma (HCC) has not been investigated in detail. The aim of this study was to clarify the cytotoxic effects of PNU-74654 against HCC and to uncover its molecular mechanism. *Materials and Methods*: HepG2 and Huh7 liver cancer cell lines were selected to determine the antitumor properties of PNU-74654. Survival of the liver cancer cells in response to PNU-74654 was assessed by cell viability assays, and the apoptosis effect of PNU-74654 was analyzed by flow cytometry and visualized by Hoechst staining. An oncology array was used to explore the underlying molecular routes of PNU-74654 action in the cells. The migration properties were examined with a wound healing assay, and western blotting was conducted to evaluate protein expression. *Results*: Treatment with PNU-74654 decreased cell viability and inhibited cell migration. The cell cycle analysis and Hoechst staining revealed an increase in the population of cells at the sub-G1 stage and apoptotic morphological changes in the nucleus. The oncology array identified 84 oncology-related proteins and a suppressed expression of Bcl-xL and survivin. Western blotting showed that PNU-74654 could interfere with cell cycle-related proteins through the NF-κB pathway. *Conclusions*: PNU-74654 shows antiproliferative and antimigration effects against HepG2 and Huh7 cells, and its antitumor activity may be attributable to its interference in cell cycle regulation and the NF-κB pathway.

## 1. Introduction

Liver cancer is an aggressive malignancy and the seventh most common neoplasm worldwide, with 905,677 new cases reported in 2020 [1]. The five-year survival rate for liver cancer is only 18%, making it the second most fatal disease in the world [2]. Hepatocellular carcinoma (HCC), the most prevalent type of liver cancer, accounts for 90% of all liver cancers [3]. The distant stage is found at the time of diagnosis in approximately 18% of HCC patients, and these patients require systemic therapy [4]. Unfortunately, even with therapy, the prognosis remains low. For example, the median overall survival was only 10 and 12 months with a sorafenib regimen in two separate clinical trials [5].

According to the National Comprehensive Cancer Network guidelines, systemic therapy should be administered to HCC patients, including those who are not candidates for liver transplantation or who suffer from metastatic disease. The recommended first-line systemic therapy regimens for HCC include a vascular endothelial growth factor (VEGF)-targeted agent plus an immune checkpoint inhibitor (ICI) or multiple tyrosine kinase inhibitors (mTKIs) alone. By contrast, the subsequent-line therapy for HCC is mainly antibodies for tyrosine kinase receptors, such as the c-MET or VEGF receptor [6]. These treatment options have been supported by complete clinical trials, and an uplifting outcome of one randomized clinical trial demonstrated that the mTKI sorafenib is an effective first-line therapy for patients with advanced HCC [7,8]. Apart from mTKIs, currently approved drugs for patients with HCC include ICIs, such as pembrolizumab and nivolumab [9,10]. However, this intractable cancer shows therapy resistance and is difficult to treat [11], so alternative therapies are needed.

One potential therapeutic target is the Wnt signaling pathway. This pathway is involved in several biological processes, as well as in embryogenesis and tumorigenesis [12]. In the canonical Wnt pathway, the Wnt protein binds to the Wnt receptor, Frizzled (FZD), to induce the β-catenin destruct complex and a phosphorylated lipoprotein receptor-related protein. This process results in stabilization and an increase in the level of β-catenin, thereby inducing nuclear translocation and, in turn, activating the Wnt-related gene [13]. Aberrations in the Wnt pathway have been observed in a variety of neoplasms, such as colorectal cancer and non-small cell lung cancer [14,15]. In patients with HCC, β-catenin is encoded by CTNNB1, and the. mutation of this gene is a frequent molecular alteration closely correlated with the abnormal Wnt signaling cascade [16]. CTNNB1 mutation causes β-catenin to escape from the destruction complex to further activate cell proliferation, cell survival, and the epithelial-to-mesenchymal transition (EMT) [12,17]. A molecular characteristic analysis has revealed that this mutation predicts refractoriness to immunotherapy; therefore, HCC with this mutation is classified in the immune-excluded class [18,19]. This genomic subtype of HCC may also influence the efficacy of sorafenib [18,20].

The Wnt pathway is a promising therapeutic target for many cancers. At the time of writing, no Wnt-targeted therapy has yet been approved, but related clinical trials are ongoing [21]. Wnt inhibitors include the PORCN inhibitor, the Wnt ligand and receptor antibodies, β-catenin or related protein inhibitors, and β-catenin target gene inhibitors [22]. In recent preclinical studies, the novel Wnt inhibiting agent PNU-74654 has shown antineoplastic ability in breast cancer and adrenocortical tumors [23,24]. PNU-74654 suppresses the Wnt pathway by disturbing the β-catenin/TCF complex and ultimately blocking the Wnt target genes [23]. However, no study has investigated the anti-cancer effect of PNU-74654 on HCC. In the present investigation, our objective was to explore the potential therapeutic effect of this Wnt pathway inhibitor on HCC. We hypothesized that PNU-74654 would have an antitumor effect against HCC.

## 2. Materials and Methods

### 2.1. Cell Cultures

Human hepatoblastoma HepG2 cells and hepatocellular carcinoma Huh7 cells were obtained from the Japanese Collection of Research Bioresources Cell Bank (BCRC, Hsinchu 300193, Taiwan). Both cell lines were maintained in Dulbecco’s Modified Eagle Medium supplemented with 10% fetal bovine serum (FBS), 100 U/mL penicillin, 100 μg/mL streptomycin, 2 g/mL NaHCO_3_, 110 mg/L sodium pyruvate, 0.1 mM NEAA, and 1000 mg/L glucose. The cells were kept in an incubator at 37 °C and supplied with 5% CO_2_.

### 2.2. Cell Viability Assay

The HepG2 and Huh7 cells were cultured overnight in 96-well plates and further treated for 24 h with PNU-74654 (MedChemExpress, Monmouth Junction, NJ, USA) at doses of 0, 50, 100, 150, 200, and 250 μM. The MTT reagent (0.5 mg/mL) was added to the wells and incubated for 3 h at 37 °C, followed by resolution in dimethyl sulfoxide. The absorbance (optical density) was measured at 570 nm with an ELISA plate reader [25,26].

### 2.3. Flow Cytometry

Cell cycle analysis and apoptosis analysis were performed using a FACSCanto^TM^ II Cell Analyzer (BD Biosciences, Franklin Lakes, NJ, USA) [25,26]. The cell lines were first cultured in a six-well plate and treated with PNU-74654 at doses of 0, 50, and 150 μM for 72 h. For analysis of the cell cycle distribution, the harvested cells were fixed with cold 70% ethanol overnight, resuspended in phosphate buffered saline (PBS) containing 4 μg/mL propidium iodide (PI) and 0.5 mg/mL RNase A, and analyzed by flow cytometry. The cell cycle distribution was assessed using FlowJo software version 10 (BD Biosciences, Franklin Lakes, NJ, USA).

### 2.4. Hoechst 33,342 Staining

Apoptosis was observed by Hoechst 33,342 staining. The cell lines (2.5 × 10^5^ cells/well) were seeded in a six-well plate and treated for 24 h with PNU-74654 at 0, 50, and 150 μM. After washing with PBS and staining with 10 μg/mL Hoechst 33,342 (Invitrogen, Carlsbad, CA, USA), apoptotic cells were detected by fluorescence microscopy (ImageXpress PICO, San Jose, CA, USA) at excitation wavelengths of 350–390 nm and emission wavelengths of 420–480 nm. The apoptotic cells were identified by their condensed and fragmented nuclei, and their percentages were calculated in five different fields at 20× magnification [25,26].

### 2.5. Wound Healing Assay

Cell migration was assessed by wound healing tests. Huh7 cells were seeded in ibidi culture inserts (Ibidi, Gräfelfing, Germany) at a density of 3.0 × 10^4^ cells per well. After 24 h, the inserts were removed, and the cells were treated with PNU-74654 at 0, 50, and 150 μM in 0.5% FBS medium. Images of the wounds were captured with a microscope at 0, 24, and 48 h after treatment. The images were analyzed using ImageJ software version 1.52a (National Institutes of Health, Bethesda, MD, USA). Each experiment was carried out in triplicate.

### 2.6. Human Oncology Array for Proteome Profiling

The proteome was profiled using the Proteome Profiler Human XL Oncology Array ARY026 (R&D Systems, Minneapolis, MN, USA), which consists of 84 human cancer-related proteins. The HepG2 and Huh7 cells were treated with and without PNU-74654 (150 μM) for 24 h, and 200 μg protein was used for each matrix, according to the manufacturer’s instructions. A horseradish peroxidase-conjugated antibody was incubated with the membranes, followed by a chemiluminescent reagent, and the cells were examined with an ImageQuant LAS4000 instrument (GE Healthcare, Marlborough, MA, USA). ImageJ software was used to analyze the integrated density of each membrane [25,26].

### 2.7. Protein Extraction and Western Blotting

The HepG2 and Huh7 cells were treated with PNU-74654 (0 and 150 μM; 48 h). The cells were washed with PBS, and the protein was extracted with RIPA buffer with protease inhibitor and phosphatase inhibitor cocktail tablets (Roche Applied Science, Mannheim, Germany) dissolved in PBS. The extract was further lysed in a −80 °C freezer and centrifuged at 13,800× *g* and 4 °C for 20 min. A Bio-Rad protein assay (Bio-Rad Laboratories Inc., Contra Costa, CA, USA) was used to determine the protein concentration in the supernatant. Protein samples (15 μg) were separated by sodium dodecyl sulfate polyacrylamide gel electrophoresis and transferred to Immobilon^TM^-P transfer membranes (Merck Millipore, Burlington, MA, USA). The membranes were blocked with 5% nonfat milk for 90 min and then incubated overnight with the following primary antibodies (diluted 1:1000): Rb (A3618, ABclonal), p-Rb (AP0117, ABclonal), CDK1 (19532-1-AP, Proteintech), CDK2 (A18000, ABclonal), Cyclin A (A7632, ABclonal), Cyclin B (A2056, ABclonal), IκBα (A11397, ABclonal), p-IκBα (AP0999, ABclonal), p50 (A6667, ABclonal), p-p50 (AP0125, ABclonal), p65 (A19653, ABclonal), and p-p65 (AP0215. ABclonal). The membranes were washed with Tris-buffered saline containing Tween 20 (TBST), incubated with secondary antibodies for 1 h. Afterwards, the membranes were washed again with TBST and treated with Immobilon^TM^ Western Chemiluminescent HRP Substrate (Merck Millipore, Burlington, MA, USA). The proteins were detected with a ImageQuant LAS4000 instrument (GE Healthcare, Marlborough, MA, USA) [25,26].

### 2.8. Statistical Analysis

All data were expressed as the mean ± standard deviation. Statistical analyses were performed using IBM SPSS software version 20.0 (Armonk, NY, USA). The student’s *t*-test was used for data analysis. All tests were two-sided, and a *p* value < 0.05 was considered statistically significant (* *p* < 0.05; ** *p* < 0.01; *** *p* < 0.001).

## 3. Results

### 3.1. PNU-74654 Inhibited Proliferation and Promoted Apoptosis in HepG2 and Huh7 Cells

**Figure 1:**
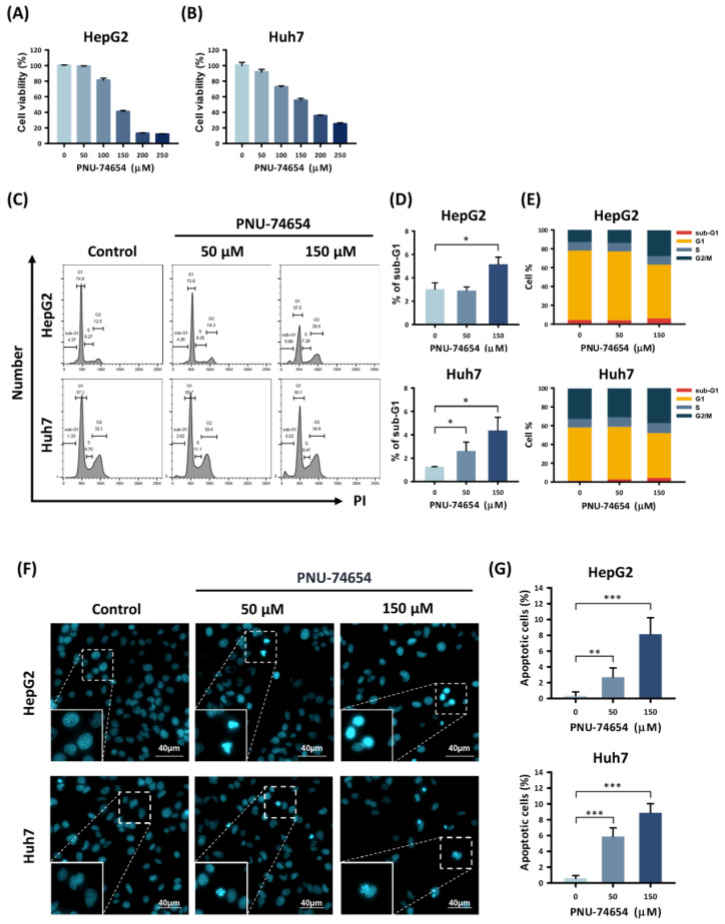
The effects of PNU-74654 on the survival of HCC cells. (**A**,**B**) Effects on cell viability, determined by viability assays in HepG2 and Huh7 cells. (**C**) Cell cycle distribution after 72 h treatment with PNU-74654 (**D**,**E**) Proportion of HCC cells in the sub-G1 cell cycle stage. (**F**) Apoptotic cells labeled by Hoechst staining. (**G**) PNU-74654 dose response of the percentages of apoptotic HCC cells. The data are presented as mean ± SD (* *p* < 0.01; ** *p* < 0.01; *** *p* < 0.001).

The MTT assay was used to assess the viability of the HepG2 and Huh7 cells following PNU-74654 treatment. PNU-74654 caused a dose-dependent reduction in cell viability (Figure 1A,B) and increased the proportion of cells in the sub-G1 cell cycle stage, with maximal effects when administered at 150 μM for 72 h (sub-G1 phase proportions: from 3.0 ± 0.6% to 5.2 ± 0.6%, *p* = 0.011, for HepG2 cell and from 1.3 ± 0.03% to 4.4 ± 1.1%, *p* = 0.041, for Huh7 cell) (Figure 1C–E). We also analyzed the proportions of HepG2 and Huh7 cells in the sub-G1 stage following a 24 h treatment with PNU-74654. The proportions of cells in all cell cycle stages are provided in Supplementary Figure S1. Cell morphology was observed by Hoechst staining and fluorescence microscopy (Figure 1F,G). The HCC cell lines showed significantly increased numbers of apoptotic cells in response to treatment with PNU-74654 (from 0.3 ± 0.5% to 8.2 ± 2.1%, *p* = 0.001 for HepG2 cell and from 0.6 ± 0.4% to 8.9 ± 1.2%, *p* = 0.001 for Huh7 cell).

### 3.2. PNU-74654 Exerted Pro-Apoptotic Effects via Bcl-xL and Survivin

**Figure 2:**
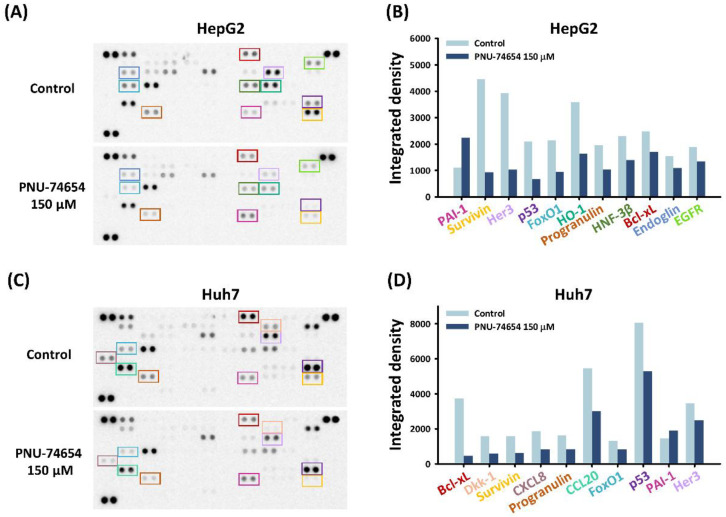
Effects of PNU-74654 on the expression of Bcl-xL and survivin. (**A**–**D**) An oncology array was conducted to analyze the underlying mechanism of the antineoplastic properties against HCC cells.

The pro-apoptotic effect of PNU-74654 was further examined by conducting a human profiler oncology array to reveal the underlying pathway in HepG2 and Huh7 cells. PNU-74654 treatment of both cell types at 150 μM downregulated the expression of the pro-apoptotic proteins Bcl-xL and surviving (Figure 2A–D). These proteins impart tumor cells with resistance to apoptosis and cancer therapies, thereby playing crucial roles in tumor progression [27,28].

### 3.3. PNU-74654 Suppressed Migration of HepG2 and Huh7 Cells

**Figure 3:**
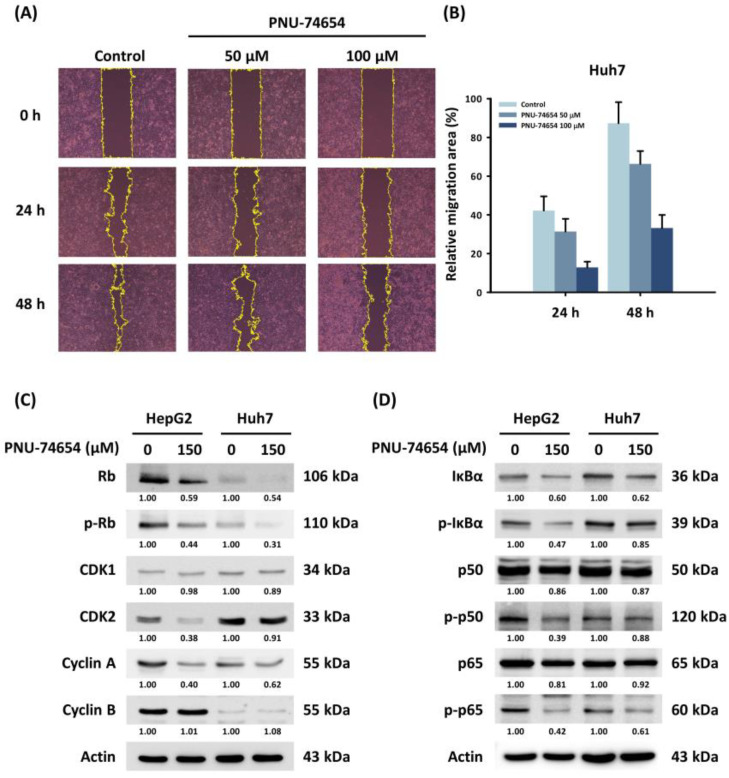
Effects of PNU-74654 on the migration of HCC cells and on the regulation of the cell cycle and the NF-κB pathway. (**A**) The migration ability of Huh7 cell, detected with a wound healing assay. (**B**) Quantification of relative migration area of Huh7 cell on 24 and 48 h. Bars depict means ± SD. (**C**,**D**) Western blots showing changes in protein expression by PNU-74654.

PNU-74654 inhibits the Wnt pathway by binding β-catenin, which acts as an important factor in cancer cell migration [23]. The wound healing assay revealed a dose-dependent reduction in cell migration in PNU-74654-treated Huh7 cells (Figure 3A,B). However, western blotting did not show a significant difference in expression of the EMT markers in response to PNU-74654.

### 3.4. PNU-74654 Exerted Its Antitumor Effect through Cell Cycle Regulation and the NF-κB Pathway

Western blotting revealed that PNU-74654 suppressed cyclin-dependent kinase 2 (CDK2) and cyclin A, which are essential regulators of cell mitosis (Figure 3C). The downregulation of CDK2/cyclin A also results in the hypo-phosphorylation of the retinoblastoma protein (Rb); thus, the expression of phosphorylated Rb (p-Rb) is inhibited [29]. The arrest of the cell cycle in HepG2 and Huh7 cells appeared to result from downregulation of the NF-κB pathway (Figure 3D) [30]. Cytochrome C, APAF1, and cleaved caspase-3 were induced by PNU-74654 treatment (Figure S2).

## 4. Discussion

This study is the first to show the antitumor effect of PNU-74654 on HCC. Here, we have demonstrated that PNU-74654 inhibits cancer cell growth by increasing the proportion of sub-G1 stage cells and promoting apoptosis. In addition, the oncology matrix identified Bcl-xL and survivin as having important roles in the pro-apoptotic effect of PNU-74654. We also showed that PNU-74654 inhibits cell growth through cell cycle regulation and the NF-κB pathway. Overall, our results support a therapeutic activity of PNU-74654, a Wnt pathway inhibitor, against HepG2 and Huh7 cells.

PNU-74654 shows suppression of cell proliferation in several types of tumors. It inhibits tumor progression by inhibiting the interaction of the TCF/β-catenin complex and disrupting the expression of genes related to cell survival, migration, and proliferation [23]. PNU-74654 also shows anti-growth activity and has a synergistic effect with 5-flourouracil in breast cancer [24]. PNU-74654 demonstrates antileukemic activity and improves the chemosensitivity of acute myeloid leukemia in mouse xenograft models [31]. PNU-74654 can also decrease cell survival and growth in the rare malignancy of feminizing adrenocortical carcinoma [32]. Consistent with these published results, our work demonstrates that PNU-74654 can also inhibit cell survival of HepG2 and Huh7 cells (Figure 1A,B).

In the present study, PNU-74654 promoted sub-G1 arrest and increased the proportion of apoptotic cells (Figure 1C–G). Further analysis revealed that decreases in the antiapoptotic proteins Bcl-xL and survivin were the underlying reasons for the apoptosis-promoting effect of PNU-74654 (Figure 2). Wnt signaling induces β-catenin translocation to the nucleus to form the β-catenin/T-cell factor transcriptional activator, which up-regulates survivin [33]. PNU-74654 augments apoptosis in adrenocortical cancer by inhibiting the TCF/β-catenin complex [23]. Disrupting or inhibiting the β-catenin/TCF complex can eventually lead to repression of survivin expression.

In patient-derived xenograft models, disrupting the TCF/β-catenin complex with other agents, such as HI-B1, also attenuates cell growth and increases apoptosis in colorectal cancer. It also negatively regulates the level of c-myc expression, thereby blocking β-catenin-driven tumorigenesis [34]. The suppression of Bcl-xL is associated with apoptotic body formation and sub-G1 arrest in HepG2 cells [35]. Apoptosis is induced in human liver cancer cells through a decrease in Bcl-xL and p-Rb due to an impairment of β-catenin [36].

The Wnt/β-catenin pathway is correlated with tumor cell migration, and our results with PNU-74654 suggest that it might inhibit HCC cell migration (Figure 3A). However, the EMT molecular route we analyzed showed no differences in response to PNU-74654. Several reports have indicated that upregulation of the Wnt pathway is correlated with the EMT and further promotes metastasis in many types of malignancies [37,38,39]. The enhanced expression of the pleomorphic adenoma gene like-2 drives the EMT through β-catenin-dependent cascades in colorectal cancer. PNU-74654 reverses this expression by suppressing the enhancement of ZEB-1 [40].

As shown in other research, PNU-74654 has a significant impact on cell cycle regulation, and the cells are trapped in the G1 phase due to increases in p53 expression and decreased expression of cyclin D and survivin [24]. In the present study, we found that the HCC cell lines were under sub-G1 arrest rather than G1 phase arrest, and our preliminary results provide a different cell cycle regulation pathway modulated by the Rb and cyclin A/CDK2 complex. The abnormal tumor suppressor protein Rb is known for its relationship with cell proliferation in human cancer [41]. The nuclear translocation of β-catenin is associated with a reduction in p16 expression, a key negative regulator that prevents Rb phosphorylation through epigenetic modulation, which then results in cell proliferation in malignant ovarian germ cell tumors [42]. This implies that the downregulation of the cell cycle pathway may be associated with the reduction in cell proliferation observed in our study. NF-κB is strongly associated with cell cycle regulation, and the best-known cell cycle regulator related to the NF-κB pathway is cyclin D1. All NF-κB-related subunits can either upregulate or downregulate cyclin D1 expression by binding to its promotor [43], in agreement with our finding that NF-κB may play a role in the regulation of the cell cycle.

In addition to changing cell cycle regulator expression, the NF-κB pathway can contribute to the anti-malignancy properties of PNU-74654, as our results demonstrate (Figure 3D). A correlation has been reported between the NF-κB pathway and cell cycle regulation, cell migration, and the regulation of inflammatory cytokines [44,45]. The activation of NF-κB by the Wnt pathway was observed in a previous study of lung adenocarcinoma cells, where progesterone membrane component 1 conferred resistance to EGFR-targeted therapy and augmented the activity of the NF-κB pathway by upregulation of the Wnt pathway [46]. Another study on breast cancer found that PNU-74654 modulated the pro-inflammatory cytokine IL-6, which is related to leukocyte recruitment [24,47]. The findings of the present study suggest that the suppression of NF-κB by PNU-74654 may inhibit tumor progression through the regulation of inflammatory cytokines.

Our study has several limitations. One is that the HepG2 and Huh7 cell lines we used cannot represent all cell variants; thus, our conclusions cannot be generalized to all subtypes of HCC. Another limitation is that no in vivo evidence was provided due to the lack of an animal model. The lack of any PNU-74654-induced changes in the expression of EMT-related proteins, which have a known involvement in cell migration, is another limitation. We also did not discuss the potential PNU-74654 effects on cancer stem cells. In addition, we have no direct evidence for a possible pathway.

## 5. Conclusions

Our preliminary results indicate that PNU-74654 has antiproliferative and antimigration properties against HepG2 and Huh7 cells. The antitumor effects of PNU-74654 appear to arise due to interference with cell cycle regulation and the NF-κB pathway.

## Data Availability

All data analyzed are included in this article, and additional information is available upon request.

## Supplementary Materials

The following supporting information can be downloaded at: https://www.mdpi.com/article/10.3390/medicina58060798/s1, Figure S1: Cell cycle distribution of HepG2 and Huh7 liver cancer cells analyzed by flow cytometry after a 24 h treatment with PNU-74654; Figure S2: Changes in pathway proteins related to cytochrome C, APAF1, and caspase signaling in HepG2 and Huh7 liver cancer cells treated with PNU-74654.
