# Supplementary material for: The Therapeutic Role of PNU-74654 in Hepatocellular Carcinoma May Involve Suppression of NF-κB Signaling
Source: Medicina (Kaunas). 2022 Jun 14;58(6):798. doi: 10.3390/medicina58060798 (PMC9228701; doi:10.3390/medicina58060798)
Supplement: Supplementary file 1 [file medicina-1750205-supplementary.pdf › medicina-1750205-supplementary.pdf]

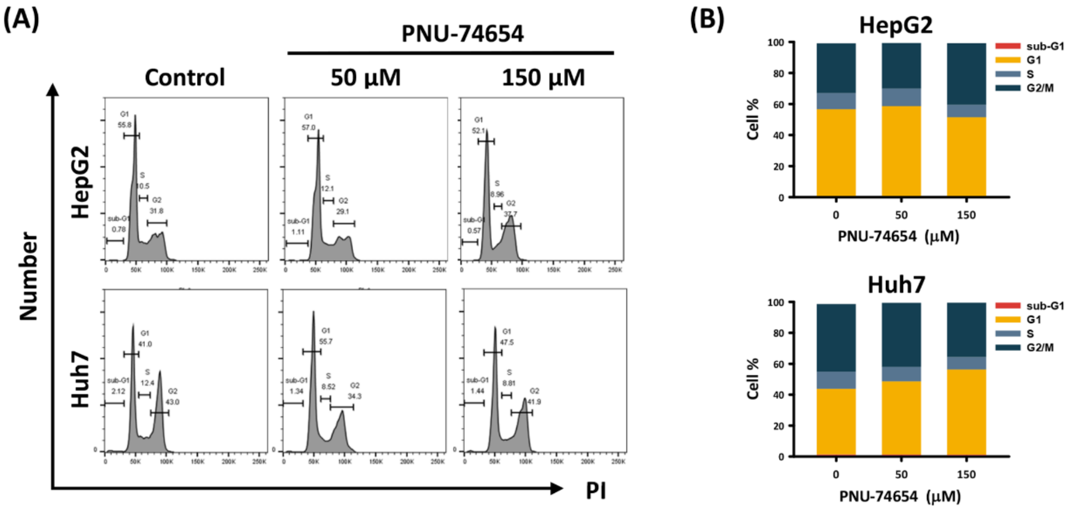

Figure S1. Cell cycle distribution of HepG2 and Huh7 liver cancer cells analyzed by flow cytometry after a 24 h treatment with PNU-74654.

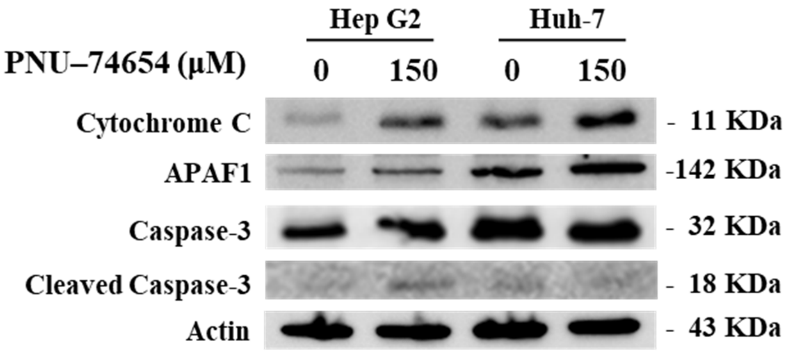

Figure S2. Changes in pathway proteins related to cytochrome C, APAF1, and caspase signaling in HepG2 and Huh7 liver cancer cells treated with PNU-74654.
